# Exploring the Interpersonal Level of Music Performance Anxiety: Online Listener’s Accuracy in Detecting Performer Anxiety

**DOI:** 10.3389/fpsyg.2022.838041

**Published:** 2022-05-10

**Authors:** Álvaro M. Chang-Arana, Anastasios Mavrolampados, Marc R. Thompson, Niklas Pokki, Mikko Sams

**Affiliations:** ^1^Brain and Mind Laboratory, Department of Neuroscience and Biomedical Engineering, Aalto University, Espoo, Finland; ^2^Department of Music, Art and Culture (MACS), University of Jyväskylä, Jyväskylä, Finland; ^3^Department of Piano, University of Arts Helsinki – Sibelius Academy, Helsinki, Finland; ^4^MAGICS, Aalto Studios, Aalto University, Helsinki, Finland

**Keywords:** music performance anxiety, multimodal perception, musical background, musical sophistication, empathic concern, shared understanding

## Abstract

Music performance anxiety (MPA) affects musicians at various stages of a performance, from its preparation until the aftermath of its delivery. Given the commonality and potentially grave consequences of MPA, it is understandable that much attention has been paid to the musician experiencing it. Consequently, we have learned a great deal about the intrapersonal level of MPA: how to measure it, treatments, experimental manipulations, and subjective experiences. However, MPA may also manifest at an interpersonal level by influencing how the performance is perceived. Yet, this has not yet been measured. This exploratory online study focuses on the listener’s perception of anxiety and compares it to the musician’s actual experienced anxiety. Forty-eight participants rated the amount of perceived anxiety of a pianist performing two pieces of contrasting difficulty in online-recital and practice conditions. Participants were presented with two stimulus modality conditions of the performance: audiovisual and audio-only. The listener’s perception of anxiety and its similarity to the musician’s experienced anxiety varies depending on variables such as the piece performed, the stimulus modality, as well as interactions between these variables and the listener’s musical background. We discuss the implications for performance and future research on the interpersonal level of MPA.

## Introduction

Music performance anxiety (MPA) affects musicians at various stages of a performance, from its preparation until the aftermath of its delivery (Kenny, 2011). Its prevalence varies from 16.5 to 60% (Fernholz et al., 2019). MPA originates from the interaction of generalized biological vulnerabilities, psychological vulnerabilities due to negative early experiences, and specific life events which establish conditioning triggers of anxiety responses (Barlow, 2000; Kenny, 2010, 2011). More recent studies focusing mostly in tertiary education students are expanding the possible dimensions involved in the development of MPA. Some of these include self-efficacy, social support (from parents, teachers, and peers), motivation, and optimism, all related to better performances or the reduction of MPA while playing (Zarza-Alzugaray et al., 2016a, 2020; Orejudo et al., 2021). Additional considerations may include the relation between the age in which musical training began and MPA (starting musical training at age 7 or younger showed lower levels of anxiety than later onsets; Zarza-Alzugaray et al., 2016b), and even the music institution students belong to Zarza-Alzugaray et al. (2016b).

In severe cases, MPA can lead to the abandonment of the otherwise promising careers (Hernández et al., 2018; Fernholz et al., 2019) or to even more dire consequences such as the development of a mood disorder (Kenny, 2011). Therefore, musicians may incur in dangerous practices, such as consuming drugs and alcohol excessively, to mitigate the debilitating symptoms of MPA (Taylor and Wasley, 2004; West, 2004; Brugués, 2011a,b; Hernández et al., 2018). Given the commonality and potentially grave consequences of MPA, it is understandable that much attention has been paid to the musician experiencing it. As a result, we have a better understanding of ways of measuring it, treatments, experimental manipulations, and its subjective experiences.

On the intrapersonal level, MPA manifests as a combination of physiological, cognitive, behavioral, and affective symptoms (as reviewed in Chang-Arana, 2015^1^, Unpublished license thesis, Chang-Arana, 2021): Physiological symptoms include accelerated heart rate, increased sweating, and higher electromyographic activity (Kim, 2008; Yoshie et al., 2008). Cognitive symptoms include catastrophic thinking, internal negative dialogues, self-destructive criticism, and a mismatch between the outcome performance and the expected level by the musician (Deniz, 2007; Kirchner et al., 2008; Brugués, 2011a). Behavioral symptoms include voluntary changes in tempo, avoidant behaviors, trembling, and in pianists contracting the shoulders (Yoshie et al., 2008, 2009; Juncos and Pona, 2018). Finally, affective symptoms include negative apprehension, subjective tension, fear of failure or guilt (Kirchner et al., 2008; Nagel, 2010; Brugués, 2011a; Kenny, 2011; Ruth et al., 2012).

On the interpersonal level, MPA can affect a musician’s performance. Performing in front of audiences or evaluators decreases performance quality scores given by juries, when comparing live vs. rehearsal performing conditions. In addition, musicians also show higher physiological arousal and increased self-reported anxiety (e.g., Brotons, 1994; LeBlanc et al., 1997; Yoshie et al., 2008, 2009; Wells et al., 2012; Kwan, 2016, Unpublished master thesis^2^). Yet, it is interesting that despite musicians’ concern about the outcome of their performances, and despite the risky measures they are willing to take to improve them little is known about whether such concerns can be detected by the listener and, if so, what drives that perception (Kwan, 2016, Unpublished master thesis, see footnote 2). It is similarly unclear whether the listener’s perception of anxiety corresponds to that of the musician. Understanding the listener can lead performers to focus on crucial performing aspects when preparing a performance. To the best of our knowledge, no previous study has addressed this question. Yet, the perceptual processes and individual variables which influence the listener’s judgment and their accuracy have been mostly unexplored (Kwan, 2016, Unpublished master thesis, see footnote 2). The listener’s judgment and the accuracy of their inferences of a performance may be affected by, for example, multimodal perception (Section “Multimodal Perception”), their musical background (Section “Musical Background and Musical Sophistication”), and dispositional empathy (Section “Dispositional Empathy”). In Sections Multimodal Perception and Musical Background and Musical Sophistication, we revisit sources first reviewed by Kwan (2016, Unpublished master thesis, see footnote 2) and complement these with other works.

### Multimodal Perception

The literature on multimodal music perception and ancillary gestures, i.e., movements which are not primarily needed to produce sounds, suggests that both observing and hearing a performance provide the listener with more information than alternative stimulus modalities (audio-only or video-only; Davidson, 1993; Vines et al., 2006; Juchniewicz, 2008; Huang and Krumhansl, 2011; Platz and Kopiez, 2012; Thompson and Luck, 2012; Vuoskoski et al., 2016). Moreover, when assessing a musical performance of a soloist, chamber ensemble or orchestra, people rely more on vision rather than hearing to judge their quality, even if the latter is believed to be the most determining factor (Tsay, 2013, 2014). The role of visual information is also important in perception of expressivity (Davidson, 1993; Vuoskoski et al., 2014), coordination (Chang et al., 2017) and interpersonal synchrony in musical interactions (Jakubowski et al., 2020), emotional tension (Wanderley et al., 2005; Vines et al., 2006), phrasing, dynamics, rubato, overall musical performance (Juchniewicz, 2008), interest on the performance (Broughton and Stevens, 2009), intensity, fluency, and professionalism (Nusseck and Wanderley, 2009; Waddell and Williamon, 2017).

Kwan (2016, Unpublished master thesis, see footnote 2) raised the argument that if a performer’s sound properties change as a result of different induced emotions, it is quite possible that MPA would affect a musician’s musical output as well as body language, and these changes could be noticeable to listeners. Previous studies have found changes in musicians’ body movements and musical outputs (e.g., articulation, timbre, and dynamics) when the musician was induced with emotions such as sadness, happiness, and anger (Dahl and Friberg, 2007; Vines et al., 2011; Van Zijl et al., 2014). Emotions have been induced by asking performers to imagine a tragic scenario or performing a piece with different expressive intentions. Therefore, can the MPA experienced by a musician be strong enough for listeners to distinguishing differences on their body movements and musical outputs across sensory modalities? According to Kwan (2016, Unpublished master thesis, see footnote 2), expressivity and performance quality were particularly impaired when listeners rated mid- and high-anxious performers in video-only conditions when compared to audiovisual and audio-only conditions. The perception of anxiety and expressivity was affected by visual stimuli (i.e., audiovisual and video-only), and performance quality was by audio stimuli. Furthermore, musical features, such as tempo, mode, or intensity, must be considered since they can also influence a perceived emotional expression (Gabrielsson and Lindström, 2010).

### Musical Background and Musical Sophistication

Musical background can have a noticeable effect on music perception. Judgments of a performance are influenced by musical training (Stanley et al., 2002; Wapnick et al., 2004; Broughton and Stevens, 2009), familiarity with the music (Flôres and Ginsburgh, 1996), and enjoyment of the piece being rated (Thompson, 2006). Musicians can detect differences in expressivity by watching the audiovisual recording or just audio recording, while non-musicians can detect differences only in the audiovisual recordings (Davidson, 2005; Huang and Krumhansl, 2011). Musicians are also capable of distinguishing differences in performance quality of ensembles of various skill levels (Geringer and Johnson, 2007; Johnson and Geringer, 2007). Some evidence also suggests that the instrument musicians play may influence their ability to detect musical quality differences of their own instrument vs. other instruments (Wapnick et al., 2004; Broughton and Davidson, 2014). Some other studies suggest that musical training might not provide advantage in detecting emotional intentions in performances (Juslin, 1997; Gabrielsson and Juslin, 2003). Kwan (2016, Unpublished master thesis, see footnote 2) first reported differences on the listener’s perception of expressivity and performance according to their musical background. But no differences in perceived anxiety were found regardless of the stimulus modality or the performance context.

However, the dichotomic categorization of musical background (non-musicians vs. musicians) has been questioned lately. Most classifications of musical background in Western societies rely on the ability of playing an instrument and the musician’s expertise in Western art music (Levitin, 2012; Müllensiefen et al., 2014; Zhang et al., 2018; Zhang and Schubert, 2019). Yet, contemporary understanding of musicianship has expanded the understanding of musical background. Therefore, we prefer using concept of musical sophistication of Müllensiefen et al. (2014, p. 2) defined as a “psychometric construct that can refer to musical skills, expertise, achievements, and related behaviors across a range of facets that are measured on different subscales.”

### Dispositional Empathy

Dispositional or trait empathy can influence the accurate detection of emotions. Higher scores of empathic concern defined as “a tendency for the respondent to experience feelings of warmth, compassion and concern for others undergoing negative experiences” (Davis, 1980, p. 6) predict higher accuracy for discriminating spontaneous from authentic laughs (Neves et al., 2018). Trait empathy factors such as perspective-taking and fantasy may be involved in the enjoyment and intensity of emotions experienced when listening to sad music (Vuoskoski and Eerola, 2012; Clark et al., 2015). Higher affective and overall empathy relate to more accurate inferences of a quartet’s expressive intentions (Wöllner, 2012). Moreover, listeners with diverse levels of musical background detect live improvisations just out of audio-tracks, while those with higher empathy levels are more sensitive (Pesquita et al., 2014).

### Aims, Research Questions, and Hypotheses

This study focuses on two aspects of the interpersonal level of MPA. First, we investigate the degree to which a listener can perceive the anxiety of a pianist. More specifically, we studied how the perceived anxiety of the pianist is influenced by the stimulus modality (audio-only vs. audiovisual), performance condition (practice vs. recital), and the piece performed (Second movement vs. Third movement), while considering the listener’s musical background. We address the following two research questions:

RQ1: How is the listener’s perceived anxiety of the player influenced by the stimulus modality, performance condition, and piece performed, while considering the listeners’ musical background?

- *Hypothesis 1.1*: There will be differences in perceived anxiety of the player depending on the stimulus modality, performance condition, and piece performed.
- *Hypothesis 1.2*: The anxiety of the player will be perceived differently depending on the listener’s musical background.

Second, we compare the listener’s perceived anxiety to the pianist’s experienced anxiety. We explore whether the difference between them is affected by the stimulus modality, performance condition, and piece performed, while considering the listener’s musical background and their disposition of empathic concern:

RQ2: Is the perceived vs. experienced anxiety influenced by the stimulus modality, performance condition, and piece performed, while considering the listener’s musical background and empathic concern?

- *Hypothesis 2.1*: There will be differences in the perceived vs. experienced anxiety depending on the stimulus modality, performance condition, and piece performed.
- *Hypothesis 2.2*: There will be differences in perceived vs. experienced anxiety depending on the listener’s musical background.
- *Hypothesis 2.3*: The differences in the perceived vs. experienced anxiety will be related to the listener’s empathic concern.

## Materials and Methods

### Ethical Approval

The aims and methods of this study have been approved by Aalto University Research Ethics Committee.

### Participants

Forty-eight participants took part in the online study [female = 30 (62.5%) male = 16 (33.3%), Trans man = 1 (2.1%), and Gender non-conforming = 1 (2.1%)]. The sample’s mean age was 30.27 (Min = 18, Max = 62, *SD* = 11.51). The subjects identified themselves as White [*n* = 33 (68.8%)], Black [*n* = 9 (18.8%)], Mixed [*n* = 3 (6.3%)], Asian [*n* = 2 (4.2%)], and Other [*n* = 1 (2.1%)]. Following Zhang and Schubert (2019), we allowed participants to self-identify as non-musicians, amateur musicians, or semiprofessional or professional musicians (Zhang et al., 2018; Zhang and Schubert, 2019). Twenty-one (43.8%) were non-musicians, 19 (39.6%) amateur musicians, and 8 (16.7%) semiprofessional or professional musician. All participants were native or fluent English speakers. Respondents were British [*n* = 27 (56.3%)], American [*n* = 8 (16.7%)], Irish [*n* = 4 (8.3%)], South African [*n* = 4 (8.3%)], and Canadian, Finn, Italian, Nigerian, and Portuguese [each one *n* = 1 (2.1%)]. Eleven participants reported playing the piano (22.9%), 26 other instruments (e.g., violin, ukulele, percussion, etc., 54.2%), and 11 none (22.9%). All participants were recruited through Prolific (Peer et al., 2017)^3^ and complete the study on the online platform Gorilla (Anwyl-Irvine, et al., 2020).^4^ At the time this article was conceived, the COVID-19 pandemic limited the possibility of recruiting participants in university facilities.

### Stimuli

The selection of the stimuli is based on the study of Kwan (2016, Unpublished master thesis, see footnote 2). In that study, musicians performed pieces of their choice in rehearsal (without an audience) and in concert (in front of an audience). The recorded performances were edited to short audio-only, video-only, and audiovisual stimuli. These were presented to observers who were instructed to rate all clips on three dimensions: expressivity of the performer, overall quality of the performance, and performer’s anxiousness. The method used in our study followed most of design of Kwan (2016, Unpublished master thesis, see footnote 2), with some modifications described below.

The first author of the present study prepared a performance of Beethoven’s piano sonata no. 6 in F major, op. 10, no. 2 from memory. The first author is a semiprofessional pianist who has been playing the piano for 17 years, received tertiary education in piano performance in Peru and Finland, and records and performs occasionally. We focused on the piano because evidence suggests musicians playing solo instruments (such as the piano) show an increase in MPA as they approach the end of their studies, when compared to orchestral musicians (e.g., violinists; Casanova et al., 2018). The piece was recorded fully in two performance conditions: practice and recital. During the practice condition, he was allowed to play the piece as many times as necessary until obtaining a satisfying recording within 1 h. No audience was present. During the recital condition, the first author invited colleagues from his department as well as other viewers with music performance background to join through Zoom. A total of 10 people witnessed the performance. The presence of the audience was intended to create the feeling of a real performance and increase the player’s anxiety, but no data was collected. The pianist wore the same clothes, played on the same piano, and recorded the performance using the same camera and microphones positioned at the same distance. The intention was to make the appearance of the practice recordings indistinguishable from the online recital recordings, so future raters would not realize the clips came from two different performance contexts.

The Second (Allegretto) and Third (Presto) movements of op. 10 no. 2 were selected given their contrasting characters in tempo, mode, and technical challenge (The complete set of stimuli can be obtained from Supplementary Material). The recordings were edited using Shotcut software (Meltytech) to obtain an approximately 1-min sample from each piece. Previous studies have also used short samples (Kwan, 2016, Unpublished master thesis, see footnote 2) because evaluations of the first sections are not that different from the entire performances (McPherson and Schubert, 2004). The face of the pianist was blurred to keep him anonymous. Each stimulus was further edited to two versions: an audiovisual and auditory-only. Although usually a video-only condition is included (Davidson, 1993), we chose not to for two reasons. First, performances where the musician plays but no sound is produced is unnaturalistic; thus, we aimed to have a more naturalistic design (Broughton and Stevens, 2009; Huang and Krumhansl, 2011). Second, participant attrition tends to be prevalent in online studies (Zhou and Fishbach, 2016), so keeping the design simpler was a way to prevent this problem.

To determine whether the performances contrasted in terms of amount of movement, we asked five professional pianists with extensive piano performance experience (mean years of experience as pianist = 28.20, *SD* = 7.36, range 18–38) to watch all four audiovisual clips of the pianist presented in counterbalanced order. After each clip, they had to rate using a 10-point Likert scale “how much did the pianist move?” (1 = “Not at all,” 10 = “Very much”). The judges perceived more movement in the Second vs. Third movement. However, there were no salient differences between two versions of the same movement (Table 1).

**Table 1:** Perceived intensity of pianist’s body movements.

	“How much did the pianist move?”			
	II movement—practice	II movement—recital	III movement—practice	III movement—recital
Pianist 1	5	5	2	2
Pianist 2	7	7	4	4
Pianist 3	8	7	2	4
Pianist 4	3	3	2	2
Pianist 5	8	7	6	3
Total scores	23	29	16	15

The inter-rater reliability of the judges was calculated through intraclass correlation (ICC). ICC estimates and their 95% CI were calculated based on a mean-rating (*k* = 5), consistency, two-way-mixed-effects model, ICC = 0.88, 95% CI [0.49, 0.99]. The judges’ inter-rater reliability is “poor” (although approaching to “moderate” reliability) to “excellent” (Koo and Mae, 2016, p. 160–161).

After the stimuli were created, the pianist rated each of them on six dimensions using a continuous scale ranging from 0 to 100. The dimensions were expressivity, control, attention, anxiety, worry, and enjoyment. The selection of dimensions was based on Kwan (2016, Unpublished master thesis, see footnote 2). However, in this study, we only report the results of the two items we used to measure anxiety:

1. How was your inner state?—This refers to how anxious the pianist was while in their playing.
2. How worried were you during the performance?—This refers to how worried or concerned was the pianist while in their playing.

### Materials

The performances were recorded using a Canon Legria HF R606 placed at 210 cm away from the middle C (C4) of the keyboard, aiming at the right side of the performer. The audio was recorded with the internal microphone of an iPhone 7 iOS 14.6 placed at 290 cm away from the keyboard. The piano was an upright Hellas Amadeus.

### Perceptual Study Procedure

Participants gave their informed consent. They were asked to complete the study in a quiet place, using headphones and their laptops/desktops. They could take as many breaks as needed between trials with unlimited duration. The aim was to allow them to complete the study in conditions similar to listening to an online recital. They were presented with the instructions and a practice trial to familiarize themselves with the task and rating system, as well as adjusting the sound volume to a comfortable level.

The participants were randomly presented with audio-only and audiovisual versions of the pieces during the practice and recital conditions. The participants did not know that the pieces came from different performance conditions. After each clip, all six evaluations were made, but we only report here the analyses of the anxiety-related responses. After completing the perceptual experiment, the participants filled the Interpersonal Reactivity Index (Davis, 1980), the Goldsmith Music Sophistication Index (Müllensiefen et al., 2014), a demographic questionnaire, and a feedback form where participants could share their experience about the study and suggest improvements for future versions.

### Questionnaires

#### Perceived Anxiety Scales

The same anxiety scales described in the Stimuli subsection were used. These were rephrased in the third person. For example, instead of asking “how was your inner state?,” participants were presented with “how was the pianist’s inner state?.” The definition of each parameter was the same as the one the pianist used (Kwan, 2016, Unpublished master thesis, see footnote 2).

#### The Interpersonal Reactivity Index

A 28-item scale, which measures four independent constructs: Fantasy, Perspective-taking, Empathic Concern, and Personal Distress. The scale has been extensively used since its creation and is under constant assessment (Wang et al., 2020). In this study, we focused our analysis on the Empathic Concern sub-scale (Davis, 1980).

#### The Goldsmith Musical Sophistication Index

A 39-item self-report scale which also includes two musicality tasks. It measures musical sophistication in general Western populations through five first-order factors (i.e., active engagement, perceptual abilities, musical training, singing abilities, and emotions) and one higher-order factor (i.e., general musical sophistication). This questionnaire does not require respondents to have a musical background, measures musical sophistication as a continuum, and has produced high internal consistency and test–retest (Müllensiefen et al., 2014).

### Data Analysis

Data were analyzed using SPSS Statistics version 27. We adjusted the *p* value following conceptualization of family-wise error of Rubin (2017, p. 273). That is, “the ‘family’ that is used to estimate the familywise error rate should consist of different tests of the same hypothesis.”

We extracted acoustic features (duration, tempo, pulse clarity, and intensity) from the four recorded pieces using the MATLAB (2021)-based MIRtoolbox (Lartillot and Toiviainen, 2007). To get the duration in seconds, we divided the total samples of each excerpt with the sampling rate (44 kHz). To detect the tempo of the four pieces, we used the *mirtempo* function which detects “periodicities in a range of beats per minutes, and choosing the maximum periodicity score for each frame separately” (Lartillot, 2021, p. 103). The pulse clarity was detected by the *mirpulseclarity* function (Lartillot et al., 2008). It “estimates the rhythmic clarity, indicating the strength of the beats estimated by the mirtempo function” (Lartillot, 2021, p. 114). Finally, to detect the intensity of the pianist’s playing on each piece, we used the *mirattackleap* function. It estimates the “amplitude difference between the beginning and the end of the attack phase (Lartillot, 2021, p. 120).

The extracted musical features are summarized in Table 2. Overall, these are very similar. The biggest difference is that the tempo (bpm) was faster in the recital conditions when compared to the practice conditions in both pieces performed.

**Table 2:** Extracted musical features.

Piece		Duration (seconds)	Tempo (bpm)	Pulse clarity	Attack leap
Second movement (Allegretto)	Practice	57.93	50.82^*^	0.30	0.16
	Recital	57.25	55.43^*^	0.32	0.12
Third movement (Presto)	Practice	94.11	130	0.32	0.16
	Recital	89.33	133.12	0.31	0.17

* The MIRtoolbox presents some difficulties when calculating the tempo of ternary metrics in pieces like the Second movement.

Original values were corrected by dividing them by 3.

#### Preliminary Analyses and Testing Assumptions

Given that the perceived anxiety and perceived worry scales seemed to be measuring the same construct, we tested if they could be combined into a single score. Each participant gave eight scores of perceived anxiety and eight scores of perceived worry. These scores were aggregated in an average to obtain two composite scores: one for perceived anxiety and another for perceived worry. We calculated the means of the composite perceived anxiety scores (*M* = 40.17, *SD* = 11.39, 95% CI [36.86, 43.47]) and the composite perceived worry scores (*M* = 36.66, *SD* = 11.39, 95% CI [33.28, 40.01]). Two Kolmogorov–Smirnov normality tests (*D*) were conducted on these composite scores. The perceived anxiety scores were approximately normally distributed, *D*(48) = 0.09, *p* = 0.20. The perceived worry scores were significantly non-normal, *D*(48) = 0.17, *p* = 0.002. Based on the central limit theorem, we considered the perceived worry scores as approximately normally distributed, given that the number of observations was above 30 (Field, 2009). A Pearson correlation analysis revealed a very strong significant association between the composite perceived anxiety scores and the composite perceived worry scores, *r* = 0.84, *p* < 0.001. Furthermore, a reliability analysis using Cronbach’s alpha revealed an internal consistency of 0.91. Based on the correlation analysis and the very high internal consistency suggesting that both composite anxiety and worry scores measure a single construct, we decided to further combine them into a single variable. From now on, it will be referred to as “perceived anxiety score.” Before participating in the study, 39 participants had not heard the Second movement and 36 participants had not heard the Third movement (*n* = 36).

We further explored the relation between music sophistication and musical background. A one-way ANOVA revealed significant differences between the scores of the GMSI general score according to the three levels of musical background (semiprofessional or professional, amateur, and non-musician), *F*(2, 47) = 15.98, *p* < 0.001, *ω*^2^ = 0.62. Planned contrasts revealed that non-musicians (*n* = 21, *M* = 61.76, *SD* = 20.26, 95% CI [52.54, 70.99]) differed significantly in their GMSI general scores from amateur and semiprofessional or professional musicians, *t*(45) = 5.64, *p* < 0.001 (one-tailed), *r* = 0.64. However, the GMSI general scores were roughly equal among amateur musicians (*n* = 19, *M* = 89.32, *SD* = 17.76, 95% CI [80.76, 97.87]) and semiprofessional or professional musicians (*n* = 8, *M* = 99.00, *SD* = 17.94, 95% CI [84.00, 114.00]), *t*(45) = 1.12, *p* = 0.231 (one-tailed), *r* = 0.18. Because amateur and semiprofessional or professional musicians did not differ in their GMSI general score, we decided to combine both groups. Thus, our variable “musical background” was recoded into two levels: non-musicians (*n* = 21) and musicians (*n* = 27).

## Results

*RQ1*: How is the listener’s perceived anxiety of the player influenced by the stimulus modality, performance condition, and piece performed, while considering the listeners’ musical background?

In RQ1, we used one test on the same hypothesis (i.e., ANOVA). Therefore, our *p* value was set to 0.05.

We conducted a 2 (Second movement vs. Third movement) × 2 (practice vs. recital) × 2 (audio-only vs. audiovisual) mixed repeated-measures ANOVA, with musical background (non-musicians vs. musicians) as between-subjects variable, and the listener’s perceived anxiety scores as dependent variable.

The perceived anxiety scores did not differ significantly between non-musicians and musicians, *F*(1, 46) = 0.52, *p* = 0.48, *η_p_*^2^ = 0.01.

The perceived anxiety scores were significantly affected by the piece being rated when ignoring the performance condition and stimulus modality, *F*(1, 46) = 6.35, *p* = 0.015, *η_p_*^2^ = 0.12. Contrasts showed that the perceived anxiety scores of the third movement (*M* = 40.24, *SE* = 1.99, 95% CI [36.23, 44.25]) were significantly higher than the perceived anxiety scores of the second movement (*M* = 35.50, *SE* = 1.76, 95% CI [31.95, 39.04]), *F*(1, 46) 6.35, *p* = 0.015, *r* = 0.35.

The perceived anxiety scores were significantly affected by the stimulus modality when ignoring the piece and performance condition, *F*(1, 46) = 4.95, *p* = 0.031, *η_p_*^2^ = 0.10. Contrasts showed that the perceived anxiety scores in the audio-only condition (*M* = 39.62, *SE* = 1.89, 95% CI [35.82, 43.42]) were significantly higher than the perceived anxiety scores in the audiovisual condition (*M* = 36.12, *SE* = 1.72, 95% CI [32.65, 39.59]), *F*(1, 46) = 4.95, *p* = 0.031, *r* = 0.31.

**Figure 1:**
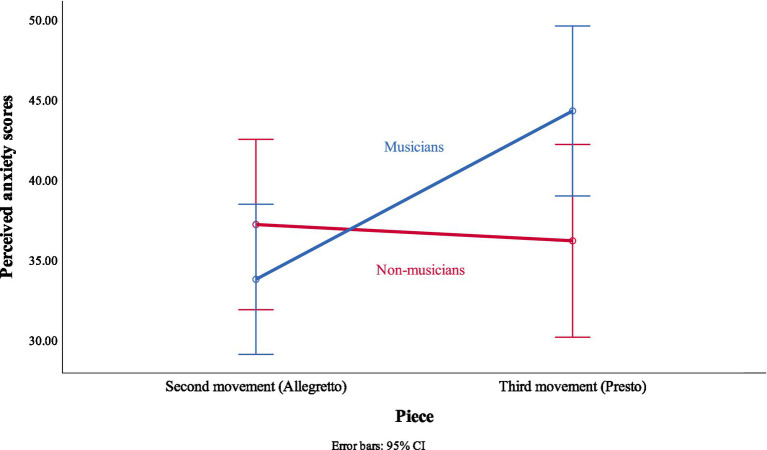
Interaction effect of piece performed and musical background.

A significant two-way interaction was found between the piece performed and the musical background of the listener, *F*(1, 46) = 9.37, *p* = 0.004, *r* = 0.41. This means that the perceived anxiety scores on each piece were different according to the musical background of the listener. The interaction figure (Figure 1) shows that the perceived anxiety scores of non-musicians were approximately the same in the Second (*M* = 37.21, *SE* = 2.64, 95% CI [31.89, 42.52]) and Third (*M* = 36.19, *SE* = 2.99, 95% CI [30.18, 42.21]) movements. However, musicians reported higher perceived anxiety scores in the Third movement (*M* = 44.30, *SE* = 2.64, 95% CI [38.99, 49.60]) when compared to the Second movement (*M* = 33.79, *SE* = 2.33, 95% CI [29.10, 38.48]).

**Figure 2:**
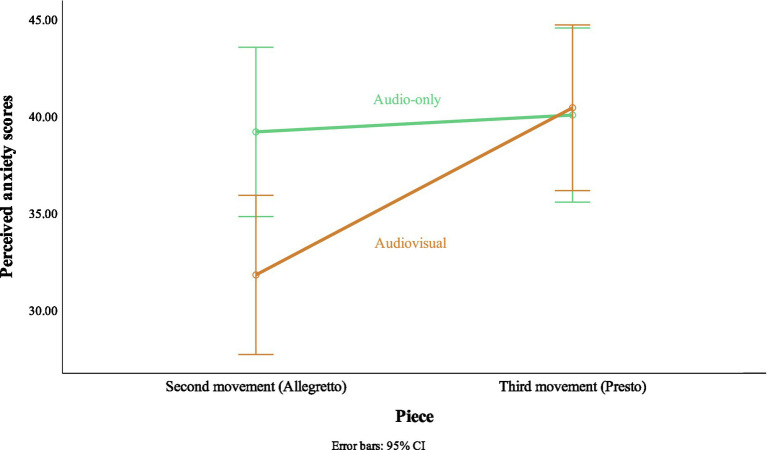
Interaction effect of piece performed and stimulus modality.

A significant two-way interaction was found between the piece performed and the stimulus modality, *F*(1, 46) = 8.66, *p* = 0.005, *r* = 0.40. This means that the perceived anxiety scores on each piece were different according to the stimulus modality. The interaction figure (Figure 2) shows that the perceived anxiety scores in the audio-only conditions were approximately the same in the Second (*M* = 39.19, *SE* = 2.17, 95% CI [34.82, 43.55]) and Third (*M* = 40.06, *SE* = 2.23, 95% CI [35.56, 44.55]) movements. However, in the audiovisual conditions, the Third movement received higher perceived anxiety scores (*M* = 40.43, *SE* = 2.12, 95% CI [36.16, 44.70]) when compared to the Second movement (*M* = 31.81, *SE* = 2.04, 95% CI [27.70, 35.92]).

*RQ2*: Is the perceived vs. experienced anxiety influenced by the stimulus modality, performance condition, and piece performed, while considering the listener’s musical background and empathic concern?

In RQ2, we used four tests on the same hypothesis (i.e., two *t*-tests, one homogeneity of regression slopes test, and one ANCOVA). Therefore, our *p* value was set to 0.013.

We calculated the difference between the listener’s perceived anxiety scores and the musician’s experienced anxiety scores. This difference was squared and then squared rooted to transform the scores into positive values. Values closer to 0 indicate higher accuracy. Conversely, values larger than 0 indicate lower accuracy. This new variable was named “anxiety-inference accuracy.”

In Table 3, we present a summary of the aggregated and averaged scores of the listener’s perceived anxiety scores and the pianist’s experienced anxiety scores. We observe that the difference between these scores is negative. That indicates that across stimuli, the listeners underestimated how anxious the pianist was feeling.

**Table 3:** Listener’s mean perceived anxiety scores and musician’s self-rated anxiety.

Piece	Performance condition and stimulus modality	Listener’s mean perceived anxiety scores	Pianist’s self-rated anxiety	Difference	*SD*	95% CI	
						Lower	Upper
Second movement (Allegretto)	Practice audio	37.95	50.00	−12.05	18.82	−17.52	−6.59
	Practice audiovisual	32.5	57.00	−24.50	18.25	−29.80	−19.20
	Recital audio	40.03	44.50	−4.47	15.51	−8.97	0.04
	Recital audiovisual	30.66	60.50	−29.84	16.98	−34.77	−24.91
Third movement (Presto)	Practice audio	40.67	44.00	−3.33	15.16	−7.73	1.07
	Practice audiovisual	40.94	44.50	−3.56	17.50	−8.64	1.52
	Recital audio	40.53	67.00	−26.47	22.49	−33.00	−19.94
	Recital audiovisual	40.86	71.50	−30.64	17.70	−35.78	−25.49

We tested the ANCOVA assumptions of independence from experimental manipulation and homogeneity of regression slopes for the anxiety–inference accuracy. Using two independent sample *t*-tests, we tested if our covariates (empathic concern, and general factor of musical sophistication) were independent from the experimental manipulation (musical background). Levene’s test suggests that the variances of empathic concern, *F*(46) = 0.01, *p* = 0.93, and musical sophistication, *F*(46) = 0.82, *p* = 0.37, are equal according to musical background. The listener’s empathic concern, *t*(46) = −0.37, *p* = 0.72, was roughly the same in the musical background groups. However, the listener’s general factor of music sophistication varied according to the musical background groups, *t*(46) = −5.49, *p* < 0.001. Thus, the general factor of music sophistication is not appropriate to use as a covariate in the analyses.

We tested if the relationship between our dependent variable (anxiety–inference accuracy) and our covariate (empathic concern) is the same in each of the musical background groups. We observed that the interaction between empathic concern and musical background was non-significant, *F*(1, 44) = 4.75, *p* = 0.211. Therefore, the assumption of homogeneity of regression slopes was met for empathic concern.

We conducted a 2 (Second movement vs. Third movement) × 2 (practice vs. recital) × 2 (audio-only vs. audiovisual) mixed repeated-measures ANCOVA, with musical background (non-musicians vs. musicians) as between-subjects variable, empathic concern as covariate, and anxiety–inference accuracy as dependent variable.

**Figure 3:**
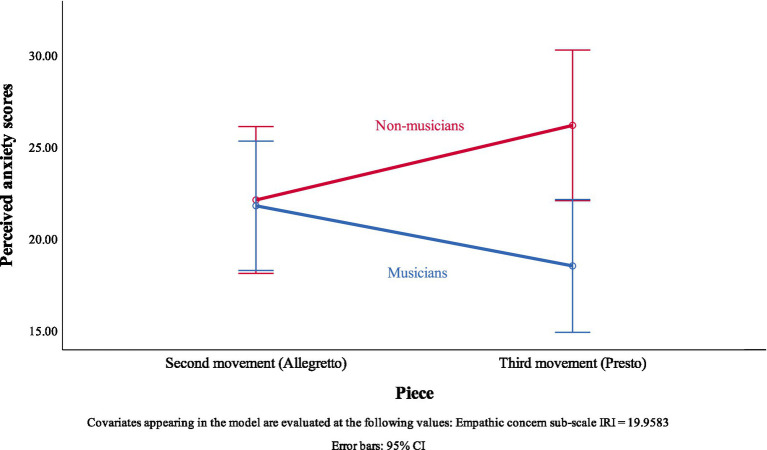
Interaction effect of piece performed and musical background.

A significant two-way interaction was found between the piece performed and the musical background of the listener, *F*(1, 45) = 7.60, *p* = 0.008, *r* = 0.38. This means that the anxiety–inference accuracy on each piece was different according to the musical background of the listener. The interaction figure (Figure 3) shows that, when adjusting for the empathic concern of the listeners, the anxiety–inference accuracy of non-musicians (*M* = 22.11, *SE* = 1.99, 95% CI [18.11, 26.11]) and musicians (*M* = 21.79, *SE* = 1.75, 95% CI [18.27, 25.32]) was approximately the same in the Second movement. However, the anxiety–inference accuracy significantly decreased for non-musicians in the Third movement (*M* = 26.17, *SE* = 2.04, 95% CI [22.07, 30.27]), while significantly increasing for musicians in the Third movement (*M* = 18.51, *SE* = 1.80, 95% CI [14.90, 22.13]).

There were no significant differences in the anxiety–inference accuracy according to musical background, even after partialling out the effect of empathic concern, *F*(1, 45) = 2.93, *p* = 0.094, *η_p_*^2^ = 0.06.

Empathic concern was not significantly related to the listener’s anxiety–inference accuracy, *F*(1, 45) = 4.75, *p* = 0.035, *η_p_*^2^ = 0.10.

## Discussion

Research in MPA mostly focuses on its intrapersonal level, that is, on the psychological processes of a musician experiencing it. As a result, we have learned much about how to measure MPA, how to treat it, its subjective experiences, how to manipulate it experimentally, and its symptoms. However, MPA has been understudied at the interpersonal level, that is, how the experienced anxiety of a musician is perceived by an audience. In this study, we explored whether listener’s perceived anxiety corresponds to the musician’s experienced anxiety. We investigated the listener’s perception through multimodal perception, while taking into account their musical background and dispositional empathic concern. Listener’s perception of performance anxiety was affected by the piece being rated (the Third movement receiving the highest perceived anxiety scores) as well as the stimulus modality (the audio-only condition receiving the highest perceived anxiety scores). Additionally, interaction effects showed that musicians perceived anxiety differently than non-musicians, and that audiovisual stimuli of the Third movement received higher perceived anxiety scores when compared to the Second movement. Listeners’ anxiety–inference accuracy was affected by the interaction of listeners’ musical background and the piece performed (Musicians showed the highest accuracy in the Third movement).

*RQ1*: How is the listener’s perceived anxiety of the player influenced by the stimulus modality, performance condition, and piece performed, while considering the listener’s musical background?

*Hypothesis 1.1*: There will be differences in perceived anxiety of the player depending on the stimulus modality, performance condition, and piece performed.

There were differences in the listener’s perceived anxiety scores according to stimulus modality and piece performed, but not according to performance condition. Furthermore, there were interactions between the piece performed and stimulus modality. Thus, we found partial support for Hypothesis 1.1. Next, we discuss these findings.

Results showed main effects of the piece performed, and stimulus modality on the perceived anxiety scores. The perceived anxiety scores were higher in the Third movement when compared to the Second movement. This corresponded with the subjective experience of the pianist, who considered the Third movement as more challenging than the Second movement. Moreover, this claim gains support due to the interaction effect we detected between the piece performed and the musical background of the listener (Figure 1). Non-musicians gave similar perceived anxiety scores regardless of the piece presented with. However, musicians reported higher perceived anxiety scores in the Third movement. This indicates that musicians may have recognized the technical difficulties demanded to play the Third movement, whereas non-musicians lacked the technical background to judge one piece as more challenging (hence more anxiety-inducing) than the other.

The listener perceived higher anxiety in the musician during the audio-only vs. audiovisual clips. This might be explained by the “protective effect” of the ancillary gestures that the listener could observe in the audiovisual stimuli. When the ancillary gestures increase, performances are judged as more expressive and they elicit positive musical experiences, such as expressiveness, interest, dynamics, phrasing, and overall musical performance (Juchniewicz, 2008; Broughton and Stevens, 2009), although some contrary evidence exists (Vines et al., 2011; Vuoskoski et al., 2016). Yet, in the study of Platz and Kopiez (2012) meta-analysis suggests that the visual component in music performance has a Cohen’s *d* medium effect size of 0.51 SD. Furthermore, the interaction between piece performed and stimulus modality (Figure 2) adds support to the protective effect of ancillary gestures. The perceived anxiety scores in audiovisual stimuli were much lower in the Second movement than in the Third movement. The difference of body movements between the Second and Third movements could explain this. In the Third movement, the pianist’s ancillary gestures were constrained, and he adopted a stiffer position in comparison with the Second movement, which allowed for more liberty of body movements. This claim is further supported by our judges who perceived more body movements in the Second movement when compared to the Third movement. In the absence of the visual component, the perceived anxiety scores in audio-only stimuli were roughly the same in both pieces.

We did not find a main effect or interaction effects of the performance condition on the perceived anxiety scores. This contradicts the subjective experience of the pianist, who felt much more anxious when performing for an online audience. The measures of this study could not capture the anxiety experience by the performer, or the anxiety cues were so subtle that the listeners were unaware of them. However, the extracted musical features (Table 2) showed that the tempo of the Second movement played in the recital condition was faster (55 bpm) than in the practice condition (50 bpm). The recital condition of the Third movement also had faster tempo (133 bpm) than the practice condition of the Third movement (130 bpm). This increase in speed may reflect the anxiety of the musician. However, the other musical features did not show major distinguishable differences.

Computational analysis of the sound properties could be an interesting additional source of information to the subjective (e.g., self-rating scales) and physiological signals (e.g., EMG, ECG, and GSR) already used to measure the manipulation of anxiety. Sound properties have shown to change depending on the performing condition or emotional state of the performers (Thompson and Luck, 2012; Van Zijl et al., 2014). Studies of social anxiety disorders suggest that changes in pitch voice could be another physiological indicator of social anxiety disorder (Weeks et al., 2012). Thus, we could find a musical equivalent in future studies with a larger sample of musicians. For example, we could investigate acoustical features such as tempo and loudness of pianists performing during high-stress conditions in comparison with lower-stress ones. Previous studies have shown that individuals with panic disorder and social phobia tend to speak faster during public speaking (Hagenaars and van Minnen, 2005; Laukka et al., 2008).

*Hypothesis 1.2*: The anxiety of the player will be perceived differently depending on the listener’s musical background.

There were no significant differences between non-musicians and musicians on their perceived anxiety scores. However, musical background did influence the perceived anxiety scores when interacting with the piece performed. Thus, we found partial support for Hypothesis 1.2.

As explained in Hypothesis 1.1, listeners with musical background may have had more tools to judge the technical demands of the two movements. Musicians perceived the Third movement as more challenging (more anxiety-inducing) than the Second movement. In contrast, non-musicians could not distinguish these technical challenges and thus gave similar perceived anxiety scores regardless of the piece presented with.

*RQ2*: Is the perceived vs. experienced anxiety influenced by the stimulus modality, performance condition, and piece performed, while considering the listener’s musical background and empathic concern?

*Hypothesis 2.1*: There will be differences in the perceived vs. experienced anxiety depending on the stimulus modality, performance condition, and piece performed.

We did not find main effects of stimulus modality, performance condition, and piece performed, on the listener’s anxiety–inference accuracy after partialling out their empathic concern (but see Supplementary Material for an alternative outcome and discussion). These results seem to conflict what the data suggest in Table 3, where the listener and the musician had quite different experiences when it comes to anxiety. The musician consistently reported experiencing anxiety levels much higher than what the listener perceived, suggesting that the listener underestimated the anxious experience of the performer. Larger differences were observed between the experienced and perceived anxiety in audiovisual vs. audio-only conditions. This supports the idea that seeing the musician performing can create a protective effect against being perceived anxious. In the absence of visual information, the listener was more accurate in their inferences. Lastly, the differences of shared understanding of anxiety observed between the Second movement and the Third movement may be driven by the ancillary gestures of the musician: The Second movement had more pronounced body gestures than the Third movement. Yet, the results of the mixed ANCOVA suggest that the anxiety–inference accuracy was not significantly affected by manipulating our main independent variables.

*Hypothesis 2.2*: There will be differences in perceived vs. experienced anxiety depending on the listener’s musical background.

There were no significant differences between non-musicians and musicians on the listener’s anxiety–inference accuracy. However, musical background did influence the listener’s anxiety–inference accuracy when interacting with the piece performed. Thus, we found partial support for Hypothesis 2.2.

In the Second movement, non-musicians and musicians had roughly the same anxiety–inference accuracy. However, in the Third movement, the accuracy of non-musicians decreased and the accuracy of musicians increased. This result can be interpreted similarly to Hypothesis 1.2. Musicians could detect the technical challenges of the Third movement and deem these as more complex than the technical challenges of the Second movement. This coincides with the pianist’s own appreciation of the higher difficulty of the Third movement in comparison with the Second movement.

Future studies should recruit participants with a stronger musical background, specifically with piano, to see how well our results generalize. In our sample, we had seven pianists who had some musical background (Only two were semiprofessional or professional musicians.). This is not enough for a valid comparison. However, this is an important question to address since previous studies report that the instrument musicians play may influence their ability to detect musical quality differences of their own vs. other instruments (Wapnick et al., 2004; Broughton and Davidson, 2014).

In our study, listeners with musical background were more accurate in detecting the experienced anxiety of the musician when playing the Third movement, and regardless of the stimulus modality. However, musical background by itself or in interaction with other variables did not make a difference in the anxiety–inference accuracy. This suggests that the pianist should not worry too much about being perceived as anxious by the audience. Similarly, during public speaking the audience do not seem to detect the speakers self-felt anxiety (Goberman et al., 2011).

Possibly, judging if a musician is anxious is not in the focus of a listener during a concert. It could be mainly a concern of the performer and goes largely unnoticed by the audience, even if specifically paid attention to. Perceiving anxiety might be harder through video recordings. Perhaps being present in the same space allows using some cues which are not transmitted through video recordings.

*Hypothesis 2.3*: The differences in the perceived vs. experienced anxiety will be related to the listeners’ empathic concern.

The listener’s empathic concern did not influence how accurately they inferred the musician’s experienced anxiety. Interestingly, this null result aligns with past research in interpersonal accuracy tests: Perceiver’s self-reported empathic dispositions do not correlate with their empathic accuracy skills (Ickes, 2001; Stueber, 2019). In Supplementary Material, we retest RQ2 without empathic concern as a covariate. Those results suggest that the listener’s anxiety–inference accuracy was affected by the performance condition (the practice condition obtained the highest accuracy) as well as the stimulus modality (the audio-only condition obtained the highest accuracy). Other interactions were detected, suggesting that the listener’s anxiety–inference accuracy varied greatly. This would align best with the claim that musicians and listeners “inhabit” different perceptual musical worlds (Schober and Spiro, 2016).

### Limitations and Future Work

Several limitations need to be pointed out. Even though, we instructed participants to complete the experiment in a quiet space and while wearing headphones, ultimately it was not possible to control adequate conditions. However, since listening to music through online platforms became quite common during the pandemic, it may be that the conditions under which participants completed the study were naturalistic. Future work could have a mixed sample of online and in-site participants completing the study and compare how results differ.

Next, we will cover limitations on the stimuli design. It may be questionable that the first author of the study was also the pianist displayed in the stimuli. Even if the first author was aware of the danger of biases, it could have been inevitable to be affected by them, particularly when it came to rate his own performances. However, this choice also allowed important learning. First, even though the pianist did feel much more anxious when performing on the online condition when compared to the practice, the metrics used by the participants did not capture this difference. Second, being an active part of this pilot study gave an experiential feeling of what future musicians would go through and thus inform best research practices with them.

We acknowledge that not including video-only conditions may have blinded us from a wider picture of multimodal perception of MPA. We hardly observe a performance where no sound is present, and thus, we decided to exclude video-only conditions from our design. This ecological validity argument has precedent on the literature (Broughton and Stevens, 2009; Huang and Krumhansl, 2011). Future studies under more controlled physical conditions may consider including video-only condition as well. However, it is worth remembering that including another stimulus condition would result in an exponential increase in the experiment’s duration, which could cause fatigue on the participants.

Not showing the facial expressions of the musician may have deprived participants of an important source of information, especially music being mostly a form of nonverbal communication. Waddell and Williamon (2017) reported low-performance quality assessment of a pianist when an aural mistake was paired with a negative facial expression. Even though the first author would have agreed to show his face, we decided to blur it because our future study is considering having more musicians. For data protection purposes, we decided to blur the faces of these future musicians. Thus, keeping the face blurred in all cases will enable some sort of comparison between studies.

Regarding the results, we emphasize that these apply to only one pianist and for our convenient sample. We believe an inherent limitation of this kind of multimodal experiment in music is its time demand. Participants took approximately 30 min to complete the ratings for one pianist being displayed in audio-only and audiovisual conditions. Adding more conditions results in longer tasks, which may discourage participants to answer conscientiously. Thus, it would be a logistical problem displaying the performances of a larger number of musicians, if what we seek is generalization. If so, measures would have to be expanded over time with the risk of participants dropping the study or responding mindlessly. Related to the results, given the nested nature of our data (several measures nested within a participant), hierarchical linear modeling would have provided a better statistical approach to analyzing our results. Yet, since we did not have missing data and the sphericity assumption was not a problem (given that our independent variables had only two levels each and at least three levels are needed for sphericity to be calculated; Field, 2009), our linear approach for examining repeated measures is viable (Heck et al., 2014).

Another limitation is that data came from judging approximately 1-min clips. The pianist and the participants did not give a judgment of the whole performance. Moreover, it does not capture how judgments evolve over time. A continuous moment-to-moment rating would be interesting as well, however, that limits the number of metrics one can use probably to one metric per watch.

As suggested in Hypothesis 1.1, extraction and analysis of musical features could be important in MPA research. We found a consistent difference in the pianist’s tempo during the recital conditions. He played faster, probably as a result of the experienced anxiety. However, listeners did not notice the difference. Thus, MIR may reveal differences, which are not detected by performers or listeners.

Future studies could control the practice effect by comparing the perception of anxiety when musicians play well-known pieces and completely new pieces. In this study, the pianist performed a well-rehearsed piece. Yet, musicians find themselves often in situations where they have little time to prepare before a performance. It is a shared believe that preparation is an important way to deal with anxiety. In the future, this could be tested by judging well-rehearsed pieces against less-rehearsed ones.

Experimental studies on MPA should be aware of the listener’s psychology. As this study suggests, the listener is not a passive evaluator of a performance. In fact, their perception is influenced by a combination of variables which should be taken into consideration in future MPA studies.

Lastly, future studies could keep a continuous measure of musical background, but still ensure that there is approximately the same number of participants of different musical backgrounds. We follow Zhang and Schubert (2019) for establishing a self-identified musical background (i.e., non-musician, amateur musician, and semiprofessional or professional musician). Thus, it would be ideal to have approximately the same number of participants per category.

## Data Availability Statement

The raw data supporting the conclusions of this article will be made available by the authors, while complying to GDPR regulations.

## Ethics Statement

The studies involving human participants were reviewed and approved by Aalto University Research Ethics Committee. The patients/participants provided their written informed consent to participate in this study.

## Author Contributions

ÁC-A obtained the ethical approval, designed and implemented the study, and wrote the manuscript. AM wrote the *MIRtoolbox* script and detected and solved the Second movement ternary-metric tempo issue. NP helped with and provided the contact of pianists for rating the body movements. MS, AM, and MT edited the manuscript. All authors provided feedback and recommendations to improve the study design. All authors contributed to the article and approved the submitted version.

## Funding

This work was supported by the Academy of Finland grant number 308431.

## Conflict of Interest

The authors declare that the research was conducted in the absence of any commercial or financial relationships that could be construed as a potential conflict of interest.

## Publisher’s Note

All claims expressed in this article are solely those of the authors and do not necessarily represent those of their affiliated organizations, or those of the publisher, the editors and the reviewers. Any product that may be evaluated in this article, or claim that may be made by its manufacturer, is not guaranteed or endorsed by the publisher.

## Footnotes

^1^Chang-Arana, Á. M. (2015). Adaptation and psychometric properties of the Kenny-Music Performance Anxiety Inventory (K-MPAI) (Unpublished license thesis). University of Lima: Peru. Retrieved from https://repositorio. ulima.edu.pe/handle/20.500.12724/1081

^2^Kwan, P. Y. (2016). The effect of music performance anxiety, context, modality and observers’ music expertise on judgment of musical performances (Unpublished master thesis). University of Jyväskylä. Available at: https://jyx.jyu.fi/handle/123456789/50620

^3^ www.prolific.co

^4^ www.gorilla.sc
