# Supplementary material for: Exploring the Interpersonal Level of Music Performance Anxiety: Online Listener’s Accuracy in Detecting Performer Anxiety
Source: Front Psychol. 2022 May 10;13:838041. doi: 10.3389/fpsyg.2022.838041 (PMC9138623; doi:10.3389/fpsyg.2022.838041)
Supplement: Supplementary file 1 [file Data_Sheet_1.pdf]

Supplementary Material

1 Supplementary Results

We present alternative results for both of our research questions. Even though we did not hypothesize that empathic concern could affect the listener's perceive anxiety, we present an ANCOVA where this relationship is tested. In RQ2, we present how results would change if we did not include empathic concern as a covariate.

RQ1: How is the listener's perceived anxiety of the player influenced by the stimulus modality, performance condition, and piece performed, while considering the listener's musical background and empathic concern?

In RQ1, we used four tests on the same hypothesis (i.e., 2 *t*-tests, 1 homogeneity of regression slopes test, and 1 ANCOVA). Therefore, our *p* value was set to .013.

We tested the ANCOVA assumptions of independence from experimental manipulation and homogeneity of regression slopes for the perceived anxiety scores, and the difference between the listener's perceived anxiety scores and the musician's experienced anxiety scores. Using two independent sample *t*-tests, we tested if our covariates (empathic concern, and general factor of musical sophistication) were independent from the experimental manipulation (musical background). Levene's test suggests that the variances of empathic concern, $F(46) = 0.01$, $p = .93$, and musical sophistication, $F(46) = 0.82$, $p = .37$, are equal according to musical background. The listener's empathic concern, $t(46) = -0.37$, $p = .72$, was roughly the same in the musical background groups. However, the listener's general factor of music sophistication varied according to the musical background groups, $t(46) = -5.49$, $p < .001$. Thus, the general factor of music sophistication is not appropriate to use as a covariate in the analyses.

We tested if the relationship between our dependent variable (perceived anxiety scores) and our covariate (empathic concern) is the same in each of the musical background groups. We observed that the interaction between empathic concern and musical background was non-significant, $F(1, 44) = 4.16$, $p = .047$. Therefore, the assumption of homogeneity of regression slopes was met for empathic concern.

We conducted a 2 (Second movement vs. Third movement) x 2 (rehearsal vs. live) x 2 (audio-only vs. audiovisual) mixed repeated-measures ANCOVA, with musical background (non-musicians vs. musicians) as between-subjects variable, empathic concern as covariate, and perceived anxiety scores as dependent variable.

A significant interaction was found between the piece performed and the musical background of the listeners, $F(1, 45) = 9.39$, $p = .004$, $r = .42$. This means that the perceived anxiety scores on each piece

were different according to the musical background of the listeners. When adjusting for the empathic concern of the listener, musicians perceived more anxiety in the Third movement (M = 44.35, SE = 2.66, 95% CI [39.00, 49.70]) than in the Second movement (M = 34.03, SE = 2.13, 95% CI [29.75, 38.31]). Conversely, non-musicians perceived approximately the same anxiety in the Second (M = 36.89, SE = 2.41, 95% CI [32.04, 41.75]) and Third (M = 36.12, SE = 3.01, 95% CI [30.05, 42.19]) movements.

Supplementary Figure 1

There were no significant differences in the perceived anxiety scores according to musical background, even after partialling out the effect of empathic concern, $F(1, 45) = 0.72$, $p = .40$, $\eta_p^2 = .02$.

Empathic concern was not significantly related to the listener's perceived anxiety scores, $F(1, 45) = 3.96$, $p = .053$, $\eta_p^2 = .08$.

RQ2: Is the perceived vs. experienced anxiety influenced by the stimulus modality, performance condition and piece performed, while considering the listener's musical background?

In RQ2, we used one test (i.e., ANOVA) on the same hypothesis (divided in 1.1 and 1.2). Therefore, our p value was set to .05.
In Supplementary Table 1, we present a summary of the aggregated and averaged scores of the listener's perceived anxiety scores and the pianist's experienced anxiety scores. We observe that the difference between these scores is negative. That indicates that across stimuli, the listener underestimated how anxious the pianist was feeling.

Supplementary Table 1

We calculated the difference between the listener's perceived anxiety scores and the musician's experienced anxiety scores. This difference was squared and then squared rooted to transform the scores into positive values. Values closer to 0 indicate higher accuracy. Conversely, values larger than 0 indicate lower accuracy. This new variable was named "anxiety-inference accuracy".

We conducted a 2 (Second movement vs. Third movement) x 2 (practice vs. recital) x 2 (audio-only vs. audiovisual) mixed repeated-measures ANOVA, with musical background (non-musicians vs. musicians) as between-subjects variable, and anxiety-inference accuracy as dependent variable.

The anxiety-inference accuracy was significantly affected by the performance condition when ignoring the piece and stimulus modality, $F(1, 46) = 29.47$, $p < .001$, $\eta_p^2 = .39$. Contrasts showed that the anxiety-inference accuracy in the practice condition (M = 18.29, SE = 1.08, 95% CI [16.11, 20.46]) was significantly higher than the anxiety-inference accuracy in the recital condition (M = 25.97, SE = 1.66, 95% CI [22.63, 29.32]), $F(1, 46) = 29.47$, $p < .001$, $r = .62$.

The anxiety-inference accuracy was significantly affected by the stimulus modality when ignoring the piece and performance condition, $F(1, 46) = 47.79$, $p < .001$, $\eta_p^2 = .51$. Contrasts showed that the anxiety-inference accuracy in the audio-only condition ($M = 18.59$, $SE = 1.29$, 95% CI [16.00, 21.18]) was significantly higher than the anxiety-inference accuracy in the audiovisual condition ($M = 25.67$, $SE = 1.34$, 95% CI [22.98, 28.37]), $F(1, 46) = 47.79$, $p < .001$, $r = .71$.

A significant two-way interaction was found between the piece performed and the musical background of the listener, $F(1, 46) = 7.83$, $p = .007$, $r = .38$. This means that the anxiety-inference accuracy on each piece was different according to the musical background of the listener. The interaction figure (Supplementary Figure 2) shows that the anxiety-inference accuracy of non-musicians ($M = 21.89$, $SE = 2.12$, 95% CI [17.62, 26.15]) and musicians ($M = 21.96$, $SE = 1.87$, 95% CI [18.20, 25.72]) was approximately the same in the Second movement. However, the anxiety-inference accuracy significantly decreased for non-musicians in the Third movement ($M = 26.08$, $SE = 2.04$, 95% CI [21.98, 30.19]), while significantly increasing for musicians in the Third movement ($M = 18.58$, $SE = 1.80$, 95% CI [14.97, 22.20]).

Supplementary Figure 2

A significant two-way interaction was found between the piece performed and the performance condition, $F(1, 46) = 37.73$, $p < .001$, $r = .67$. This means that the anxiety-inference accuracy on each piece was different according to the performance condition. The interaction figure (Supplementary Figure 3) shows that the anxiety-inference accuracy in the Second movement were approximately the same in the practice ($M = 22.28$, $SE = 1.72$, 95% CI [18.83, 25.73]) and recital ($M = 21.57$, $SE = 1.61$, 95% CI [18.33, 24.82]) conditions. However, the anxiety-inference accuracy decreased for non-musicians in the Third movement ($M = 26.08$, $SE = 2.04$, 95% CI [21.98, 30.19]), while increased for musicians in the Third movement ($M = 18.58$, $SE = 1.80$, 95% CI [14.97, 22.20]).

Supplementary Figure 3

A significant two-way interaction was found between the piece and stimulus modality, $F(1, 46) = 20.35$, $p < .001$, $r = .55$. This means that the anxiety-inference accuracy on each piece was different according to the stimulus modality. The interaction figure (Supplementary Figure 4) shows that the anxiety-inference accuracy in the Second movement was significantly higher in the audio-only condition ($M = 15.81$, $SE = 1.31$, 95% CI [13.18, 18.44]) when compared to the audiovisual condition ($M = 28.04$, $SE = 1.89$, 95% CI [24.23, 31.84]). In the Third movement, the anxiety-inference accuracy in the audio-only condition decreased ($M = 21.36$, $SE = 1.65$, 95% CI [18.05, 24.68]), while the anxiety-inference accuracy in the audiovisual condition increased ($M = 23.30$, $SE = 1.43$, 95% CI [20.43, 26.17]). Overall, anxiety-inference accuracy was higher for the audio-only condition and lower for the audiovisual condition across pieces.

Supplementary Figure 4

A significant two-way interaction was found between the performing condition and stimulus modality, $F(1, 46) = 4.75, p = .034, r = .31$. This means that the anxiety-inference accuracy on each performance condition was different according to the stimulus modality. The interaction figure (Supplementary Figure 5) shows that the anxiety-inference accuracy in the practice condition was more accurate in the audio-only condition (*M* = 16.02, *SE* = 1.05, 95% CI [13.90, 18.13]) when compared to the audiovisual condition (*M* = 20.56, *SE* = 1.50, 95% CI [17.54, 23.57]). In the recital condition, the anxiety-inference accuracy in the audio-only condition decreased (*M* = 21.16, *SE* = 1.82, 95% CI [17.49, 24.83]), as well as the anxiety-inference accuracy in the audiovisual condition (*M* = 30.78, *SE* = 1.89, 95% CI [26.97, 34.59]) when compared to the practice condition. Overall, anxiety-inference accuracy was higher for the audio-only condition and lower for the audiovisual condition playing conditions.

Supplementary Figure 5

Lastly, a three-way interaction was found between the piece, the performing condition, and stimulus modality, $F(1, 46) = 5.47, p = .024, r = .33$. In the Second movement (Supplementary Figure 6A), the anxiety-inference accuracy improved when audio-only stimuli of the recital condition were presented (*M* = 12.90, *SE* = 1.41, 95% CI [10.07, 15.73]), when compared to when audio-only stimuli of the practice condition were presented, (*M* = 18.73, *SE* = 1.80, 95% *CI* [15.11, 22.35]). Also in the Second movement, the anxiety-inference accuracy decreased when audiovisual stimuli of the recital condition were presented (*M* = 30.25, *SE* = 2.36, CI 95% [25.50, 34.99]), when compared to when audiovisual stimuli of the practice condition were presented, (*M* = 25.83, *SE* = 2.33, CI 95% [21.14, 30.51]). In the Third movement (Supplementary Figure 6B), the anxiety-inference accuracy decreased in the recital condition in comparison to the practice condition, regardless of the stimulus modality. Overall, anxiety-inference accuracy was higher for the audio-only condition and lower for the audiovisual condition in both pieces and for both playing conditions.

Supplementary Figures 6A and 6B

2 Discussion of supplementary results

Research in MPA mostly focuses on its intrapersonal level. That is, on the psychological processes of a musician experiencing it. As a result, we have learned much about how to measure MPA, how to treat it, its subjective experiences, how to manipulate it experimentally, and its symptoms. However, MPA has been understudied at the interpersonal level. That is, how the experienced anxiety of a musician is perceived by an audience. In this study we explored whether the listener's perceived anxiety corresponds to the musician's experienced anxiety. We investigated the listener's perception through multimodal perception, while taking into account their musical background and dispositional empathic concern. The listener's perception of performance anxiety was affected by the piece being rated (the Third movement receiving the highest perceived anxiety scores) when interacting with the listener's musical background. Only musicians perceived anxiety differently depending on the piece being rated: the Third movement received higher perceived anxiety scores when compared to the Second movement. Non-musicians perceived anxiety similarly in both movements. The listener's anxiety-inference accuracy was affected by the performance condition (the practice condition obtained the highest accuracy) as well as the stimulus modality (the audio-only condition obtained the highest accuracy). Other interactions were detected, suggesting that the listener's anxiety-inference accuracy varied greatly.

2.1 RQ1: How is the listener's perceived anxiety of the player influenced by the stimulus modality, performance condition, and piece performed, while considering the listener's musical background and empathic concern?

2.1.1 Hypothesis 1.1: There will be differences in perceived anxiety of the player depending on the stimulus modality, performance condition, and piece performed.

Contrarily to what is usually reported in the multimodal perception literature, the stimulus modality did not affect significantly how the listeners perceived anxiety. When the ancillary gestures increase, performances are judged as more expressive and they elicit positive musical experiences, such as expressiveness, interest, dynamics, phrasing, and overall musical performance (Broughton & Stevens, 2009; Juchniewicz, 2008). Platz and Kopiez (2012) meta-analysis suggest that the visual component in music performance has a Cohen's *d* medium effect size of 0.51 standard deviations. Although some contrary evidence exists (Vines et al., 2011; Vuoskoski et al., 2016). Yet, the studies on multimodal perception reviewed in this study did not take into account empathic concern or other factors of empathy in their design.

We did not find a main effect or interaction effects of the performance condition on the perceived anxiety scores. This contradicts the subjective experience of the pianist, who felt much more anxious when performing for an online audience. The measures of this study could not capture the anxiety experience by the performer, or the anxiety cues were so subtle that the listeners were unaware of them. However, the extracted musical features (see Supplementary Table 2) showed that the tempo of the

Second movement played in the recital condition was faster (55 bpm) than in the practice condition (50 bpm). The recital condition of the Third movement also had faster tempo (133 bpm) than the practice condition of the Third movement (130 bpm). This increase in speed may reflect the anxiety of the musician. However, the other musical features did not show major distinguishable differences. Computational analysis of the sound properties could be an interesting additional source of information to the subjective (e.g., self-rating scales) and physiological signals (e.g., EMG, ECG, GSR) already used to measure the manipulation of anxiety. Sound properties have shown to change depending on the performing condition or emotional state of the performers (Thompson & Luck, 2012; Van Zijl et al., 2014). Studies of social anxiety disorders suggest that changes in pitch voice could be another physiological indicator of social anxiety disorder (Weeks et al., 2012). Thus, we could find a musical equivalent in future studies with a larger sample of musicians. For example, we could investigate acoustical features such as tempo and loudness of pianists performing during high-stress conditions in comparison to lower stress ones. Previous studies have shown that individuals with panic disorder and social phobia tend to speak faster during public speaking (Hagenaars & van Minnen, 2005, Laukka et al., 2008).

2.1.2 Hypothesis 1.2: The anxiety of the player will be perceived differently depending on the listener's musical background.

The interaction between the piece performed and the musical background shows that non-musicians gave similar perceived anxiety scores regardless of the piece presented with. However, musicians reported higher perceived anxiety scores in the Third movement. This indicates that musicians may have recognized the technical difficulties demanded to play the Third movement, whereas non-musicians lacked the technical background to judge one piece as more challenging (hence more anxiety-inducing) than the other. This corresponded with the subjective experience of the pianist, who considered the Third movement as more challenging than the Second movement.

2.1.3 Hypothesis 1.3: The perception of the player's anxiety will be related to the listener's empathic concern.

Empathic concern was not significantly related to the listener's perceived anxiety scores. Possibly, judging if a musician is anxious is not in the focus of a listener during a concert. It could be mainly a concern of the performer and goes largely unnoticed by the audience, even if specifically paid attention to.

2.2 RQ2: Is the perceived vs. experienced anxiety influenced by the stimulus modality, performance condition and piece performed, while considering the listener's musical background?

2.2.1 Hypothesis 2.1: There will be differences in the perceived vs. experienced anxiety depending on the stimulus modality, performance condition, and piece performed.

There are differences in the listener's anxiety-inference accuracy according to performance condition and stimulus modality. No main effect of the piece performed was found. Yet, we detected interactions between piece performed and stimulus modality, between performance condition and stimulus

modality, and a triple interaction between piece performed, performance condition and stimulus modality. Thus, we found partial support for Hypothesis 2.1.

Results show a main effect of performance condition on the anxiety-inference accuracy. The listener's accuracy was higher in the practice condition when compared to the recital condition. Supplementary Table 1 indicates that the decrease in accuracy was due to the pianist reporting higher experienced anxiety scores during the recital condition when compared to the practice condition. However, the change in the pianist's anxiety experience seemed unnoticeable for listeners.

A main effect of stimulus modality on the anxiety-inference accuracy was detected. Accuracy was higher in audio-only conditions when compared to the audiovisual conditions. This further supports the idea that seeing the musician performing can create a protective effect against being perceived anxious. In the absence of visual information, listeners were more accurate in their inferences.

The piece performed interacted with the stimulus modality on the listener's anxiety-inference accuracy (Supplementary Figure 4). Accuracy in the Second movement was higher in the audio-only condition when compared to the audiovisual condition. Again, probably explained by the larger body movements of the pianist during the performance of this movement. The accuracy in the Third movement described a contrary converging trend for both stimulus modalities: the accuracy in the audio-only condition decreased and increased in the audiovisual condition. Yet, it is interesting that, overall, there was higher anxiety-inference accuracy in the audio-only stimuli when compared to the audiovisual condition.

Regarding the interaction between performing condition and stimulus modality, it should be taken with precaution. The interaction graph (Supplementary Figure 5) seems to describe parallel lines and the p value obtained here ($p = .034$) was not as low as the ones obtained in other tests of RQ2. Yet, it seems that the accuracy in the practice condition was higher in the audio-only condition when compared to the audiovisual condition. During the recital condition, the accuracy decreased in the audio-only condition.

Lastly, we interpret the three-way interaction between piece performed, performing condition, and stimulus modality. The interaction graphs (Supplementary Figures 6A and 6B) show clear differences in the anxiety-inference accuracy. In the Second movement, accuracy improved when audio-only stimuli of the recital condition were presented, when compared to when audio-only stimuli of the practice condition were presented. Continuing with the Second movement, accuracy decreased when audiovisual stimuli of the recital condition were presented, when compared to when audiovisual stimuli of the practice condition were presented. In the third movement, accuracy decreased in recital in comparison to the practice condition, regardless of stimulus modality. Overall, accuracy was higher for the audio-only condition and lower for the audiovisual condition in both pieces and for both playing conditions.

The many discrepancies of the listener-perceived and musician-experienced anxiety suggest that the musician and the listeners "inhabit" different perceptual musical worlds (Schober & Spiro, 2016). As

shown in Supplementary Table 1, the musician reported anxiety levels much higher than the listener consistently. Perhaps the pianist's concern to appear anxious pertained to him but not to the listener. Listeners may approach a performance for other reasons other than identifying if the performer is anxious.

2.2.2 Hypothesis 2.2: There will be differences in perceived vs. experienced anxiety depending on the listener's musical background.

There were no significant differences between non-musicians and musicians on the listener's anxiety-inference accuracy. However, musical background did influence the listener's anxiety-inference accuracy when interacting with the piece performed. Thus, we found partial support for Hypothesis 2.2.

In the Second movement, non-musicians and musicians had roughly the same anxiety-inference accuracy. However, in the Third movement, the accuracy of non-musicians decreased and the accuracy of musicians increased. This result can be interpreted similarly to Hypothesis 1.2. Musicians could detect the technical challenges of the Third movement and deem these as more complex than the technical challenges of the Second movement. This coincides with the pianist's own appreciation of the higher difficulty of the Third movement in comparison to the Second movement.

Future studies should recruit participants with a stronger musical background, specifically with piano, to see how well our results generalize. In our sample we had seven pianists who had some musical background (only two were semi-professional or professional musicians). This is not enough for a valid comparison. However, this is an important question to address since previous studies report that the instrument musicians play may influence their ability to detect musical quality differences of their own vs. other instruments (Broughton & Davidson, 2014; Wapnick et al., 2004).

In our study, listeners with musical background were more accurate in detecting the experienced anxiety of the musician when playing the Third movement, and regardless of the stimulus modality. However, musical background by itself or in interaction with other variables did not make a difference in the anxiety-inference accuracy. This suggests that the pianist should not worry too much about being perceived as anxious by the audience. Similarly, during public speaking the audience do not seem to detect the speakers self-felt anxiety (Goberman et al., 2011).
Possibly, judging if a musician is anxious is not in the focus of a listener during a concert. It could be mainly a concern of the performer and goes largely unnoticed by the audience, even if specifically paid attention to. Perceiving anxiety might be harder through video recordings. Perhaps being present in the same space allows using some cues which are not transmitted through video recordings.

2.3 Supplementary References

Broughton, M. C. & Davidson, J. W. (2014). Action and familiarity effects on self and other expert musician's Laban effort-shape analyses of expressive bodily behaviors instrumental music performance: A case study. *Frontiers in Psychology, 5*. DOI: 10.3389/fpsyg.2014.01201

Broughton, M. C. & Stevens, C. (2009). Music, movement and marimba: an investigation of the role of movement and gesture in communicating musical expression to an audience. *Psychology of Music*, *37*(2), 137–153. Retrieved from https://doi.org/10.1177/0305735608094511

Goberman, A. M., Hughes, S., & Haydock, T. (2011). Acoustic characteristics of public speaking: Anxiety and practice effects. *Speech Communication, 53*, 867-876. DOI:10.1016/j.specom.2011.02.005

Hagenaars, M. A. & van Minnen, A. (2005). The effect of fear on paralinguistic aspects of speech in patients with panic disorder with agoraphobia. *Anxiety Disorders, 19*, 521-537. Retrieved from https://doi.org/10.1016/j.janxdis.2004.04.008

Juchniewicz, J. (2008). The influence of physical movement on the perception of musical performance. *Psychology of Music*, *36*(4), 417–427. Retrieved from https://doi.org/10.1177/0305735607086046

Laukka, P., Linnman, C., Åhs, F., Pissiota, A., Frans, Ö., Faria, V., Michelgråd Å., Appel, L., Fredikson, M., & Furmark, T. (2008). In a nervous voice: Acoustic analysis and perception of anxiety in social phobics' speech. *Journal of nonverbal behavior, 32*, 195-214. Retrieved from https://doi.org/10.1007/s10919-008-0055-9

Platz, F. & Kopiez, R. (2012). When the eye listens: A meta-analysis of how audio-visual presentation enhances the appreciation of music performance. *Music Perception*, *30*(1), 71–83. Retrieved from https://doi.org/10.1525/mp.2012.30.1.71

Schober, M. F. & Spiro, N. (2016). Listeners' and performers' shared understanding of jazz improvisations. *Frontiers in Psychology*, *7*, 1–20. Retrieved from https://doi.org/10.3389/fpsyg.2016.01629

Thompson, M. R. & Luck, G. (2012). Exploring relationships between pianists' body movements, their expressive intentions, and structural elements of the music. *Musicae Scientiae, 16*(1), 19-40. DOI:10.1177/1029864911423457

Van Zijl, A. G. W., Toiviainen, P., & Luck, G. (2014). The sound of emotion: The effect of performers'experienced emotions on auditory performance characterist ics. *Music Perception, 32*(1), 33-50. Retrieved from https://doi.org/10.1525/mp.2014.32.1.33

Vines, B. W., Krumhansl, C. L., Wanderley, M. M., Dalca, I. M., & Levitin, D. J. (2011). Music to my eyes: Cross-modal interactions in the perception of emotions in musical performance. *Cognition, 118,* 157–170. Retrieved from http://dx.doi.org/10.1016/j.cognition.2010.11.010

Vuoskoski, J. K., Gatti, E., Spence, C., & Clarke, E. F. (2016). Do visual cues intensify the emotional responses evoked by musical performance? A psychophysiological investigation. *Psychomusicology: Music, Mind, and Brain*, *26*(2), 179–188. Retrieved from https://doi.org/10.1037/pmu0000142

Wapnick, J., Ryan, C., Lacaille, N., & Darrow, A. A. (2004). Effects of selected variables on musicians' ratings of high-level Piano performances. *International Journal of Music Education*, *22*(1), 7–20. Retrieved from https://doi.org/10.1177/0255761404042371

Weeks, J. W., Lee, C-Y., Reilly, A. R., Howell, A. N., France, C., Kowalsky, J. M., & Bush, A. (2012). "The sound of fear": Assessing vocal fundamental frequency as a physiological indicator of social anxiety disorder. *Journal of Anxiety Disorders, 26*, 811-822. Retrieved from http://dx.doi.org/10.1016/j.janxdis.2012.07.005

3 Supplementary Figures

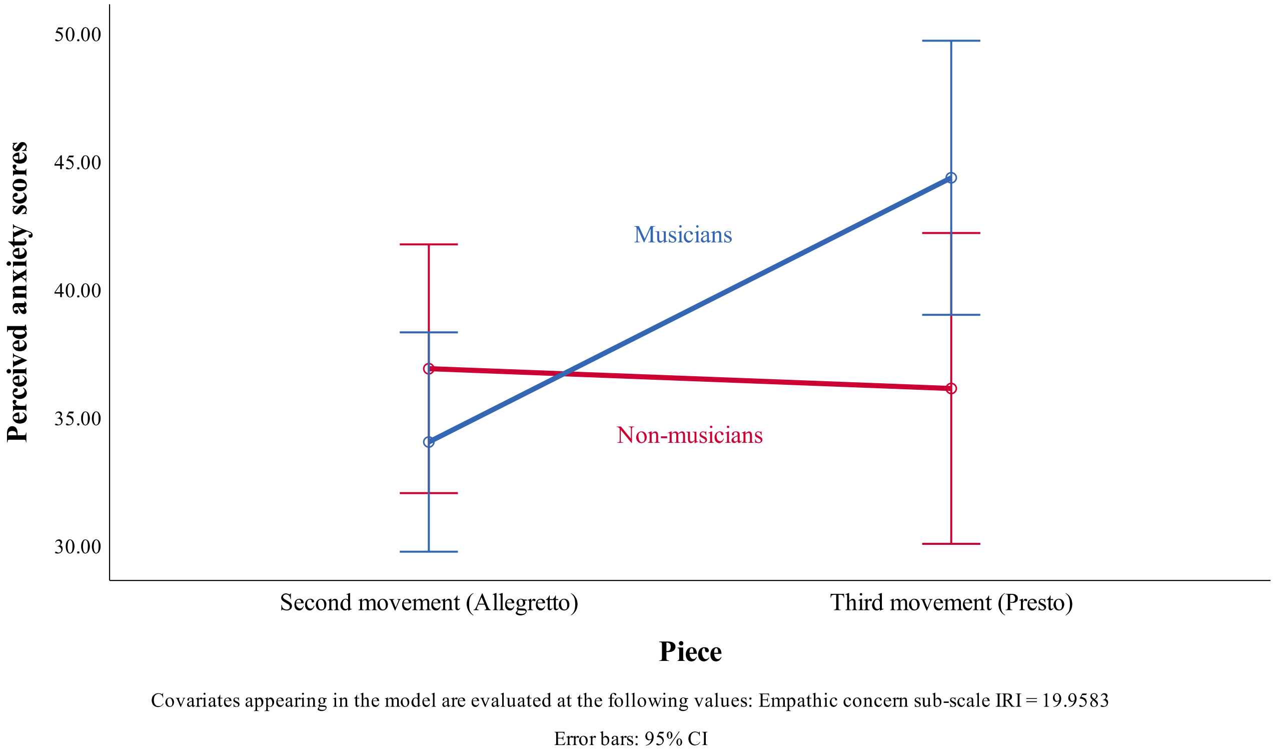

Supplementary Figure 1. Interaction effect of piece performed and musical background.

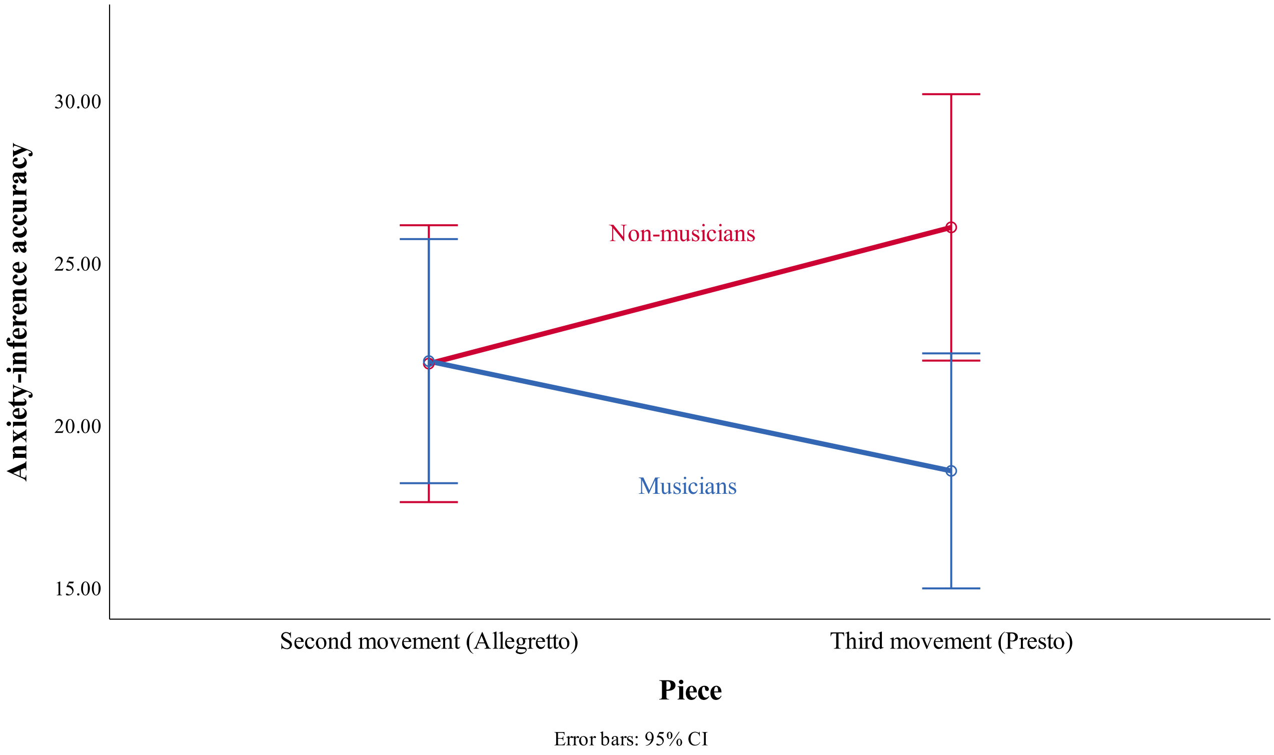

Supplementary Figure 2. Interaction effect of piece performed and musical background.

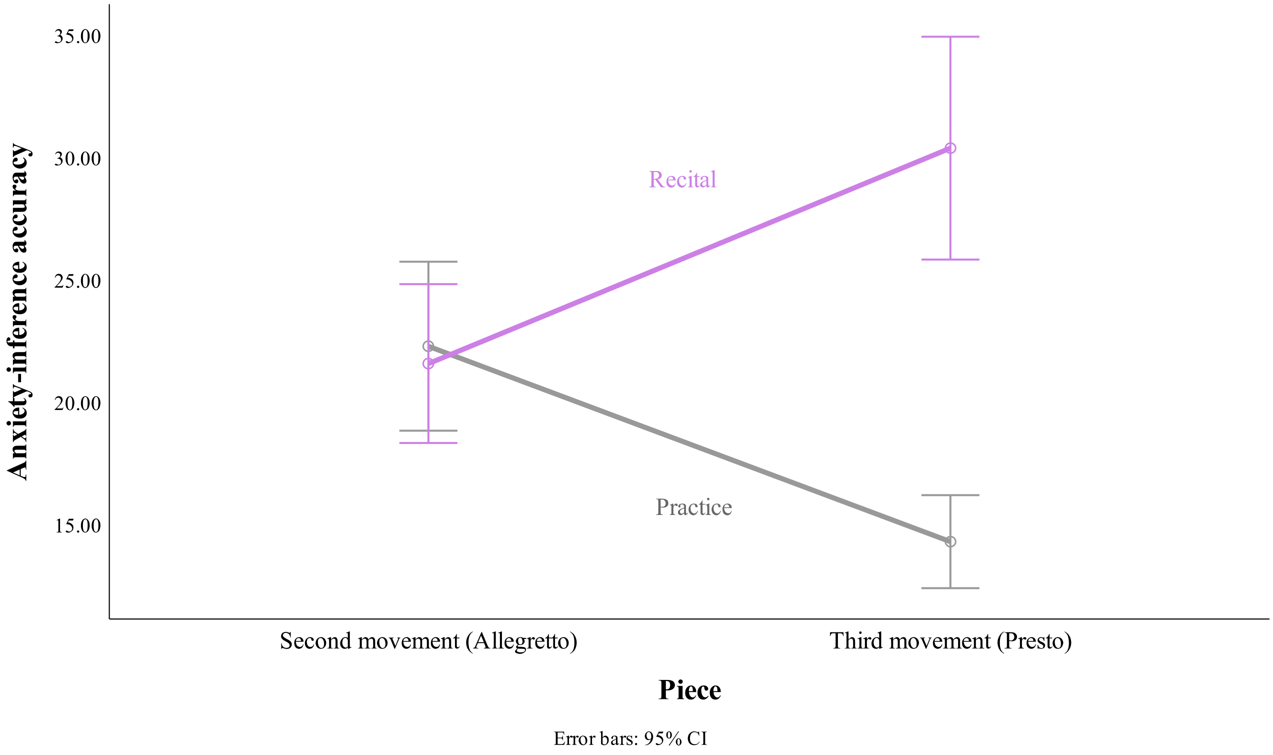

Supplementary Figure 3. Interaction effect of piece performed and performance condition.

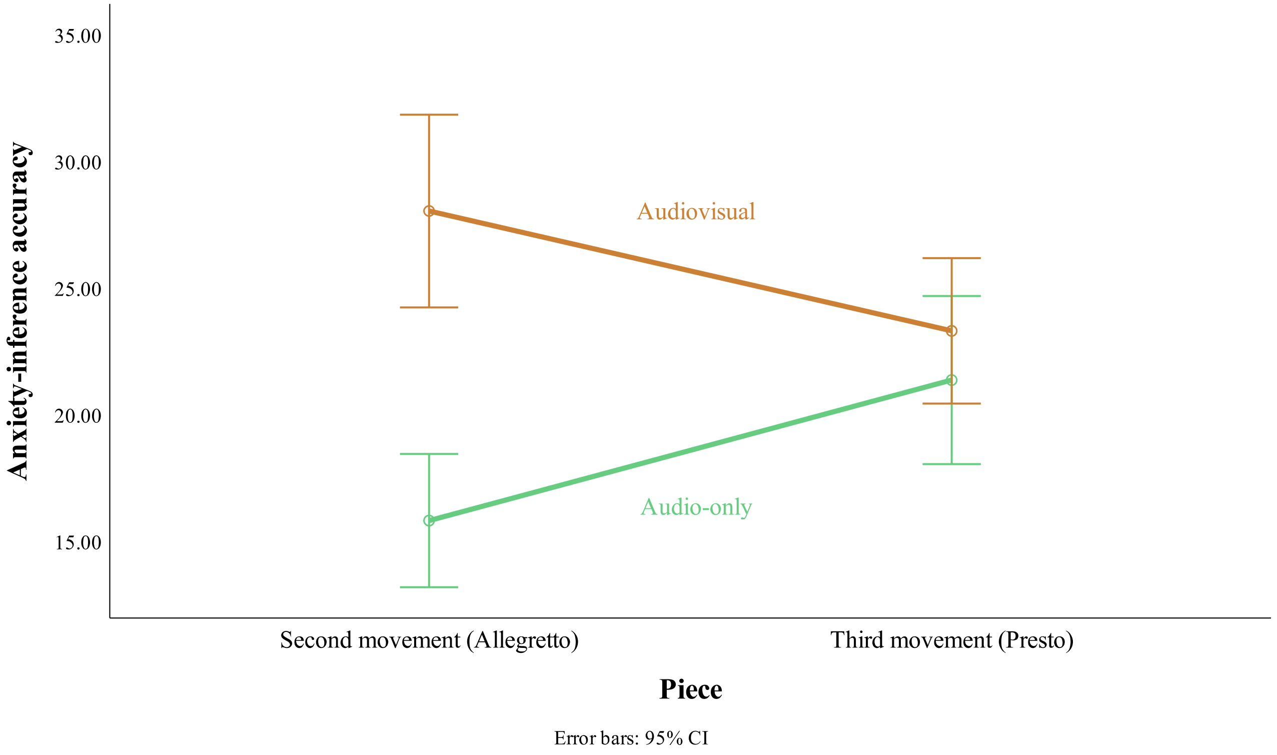

Supplementary Figure 4. Interaction effect of piece performed and stimulus modality.

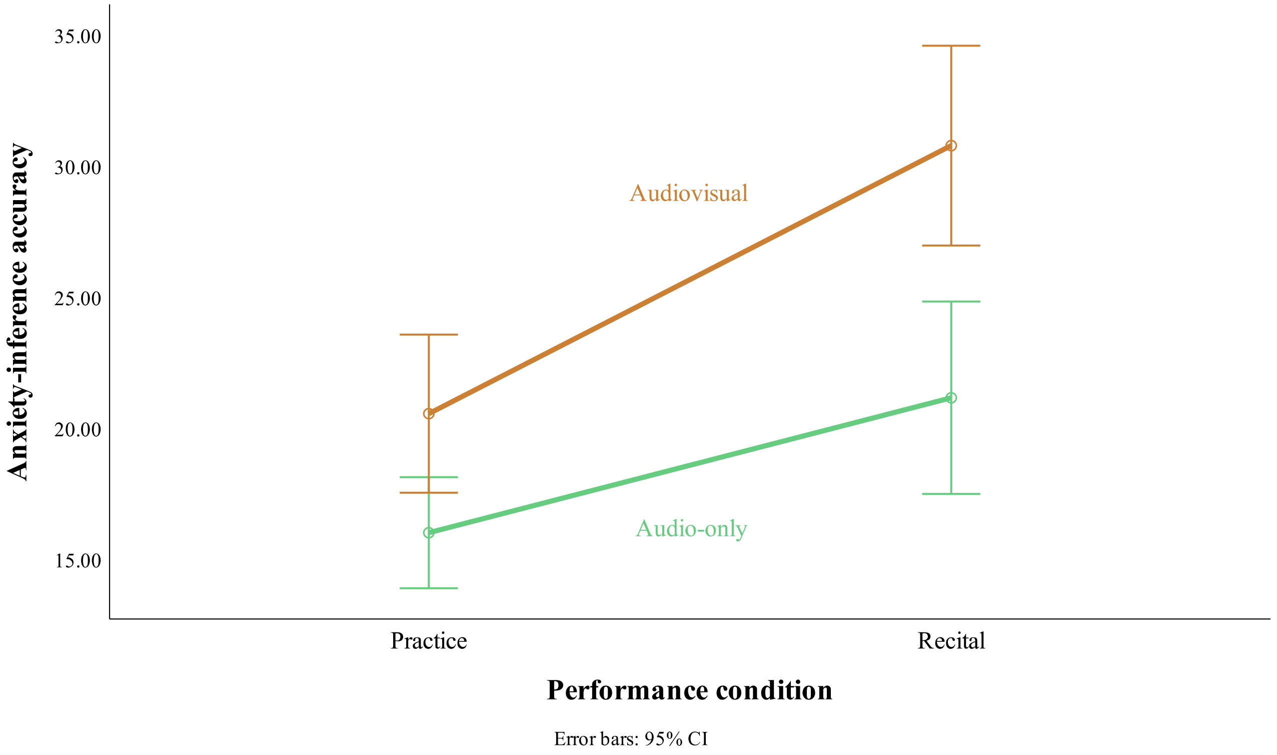

Supplementary Figure 5. Interaction effect of performance condition and stimulus modality.

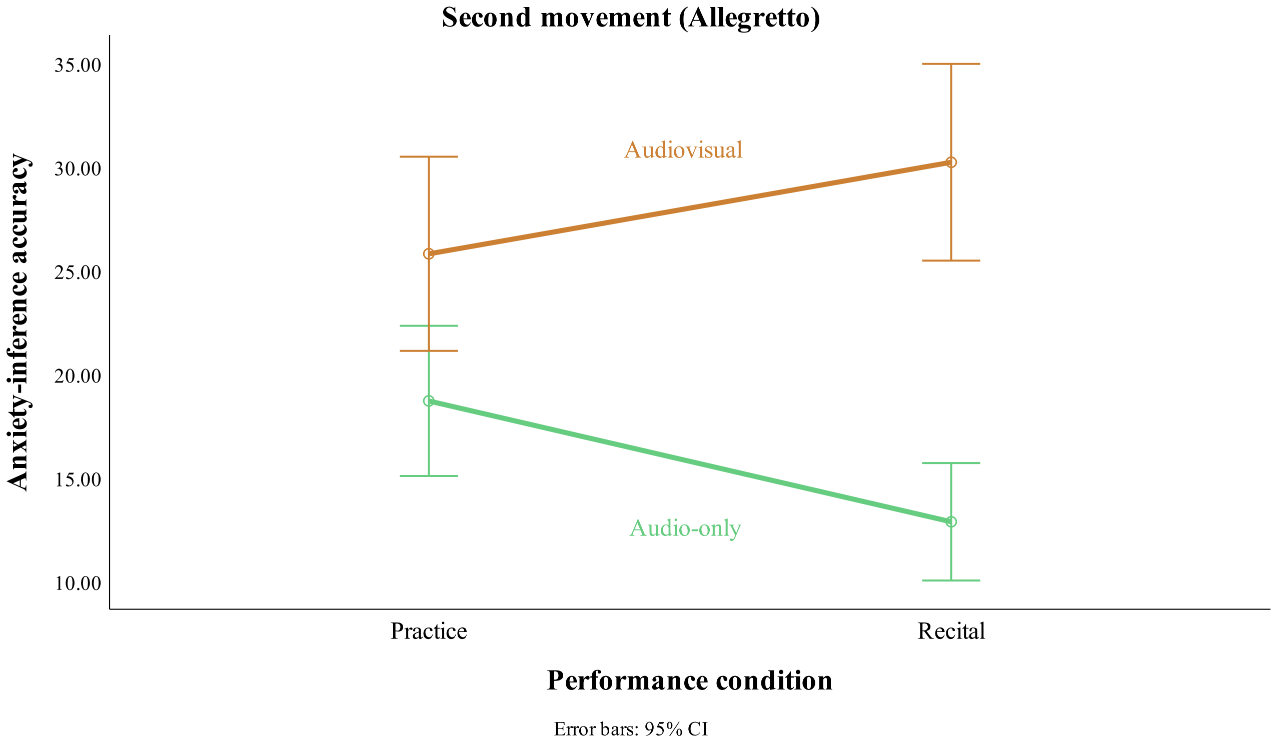

Supplementary Figure 6A. Interaction effect of piece performed (second movement), performance condition, and stimulus modality.

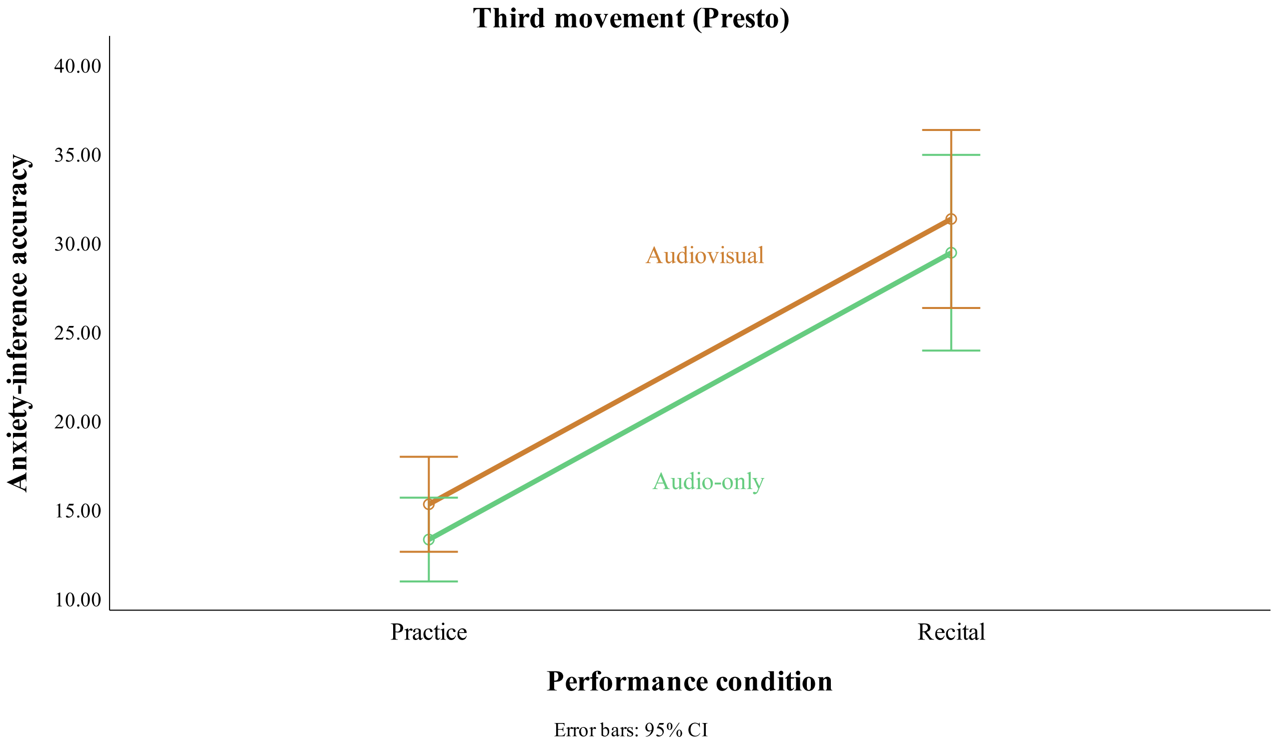

Supplementary Figure 6B. Interaction effect of piece performed (third movement), performance condition, and stimulus modality.

4 Supplementary Tables

Supplementary Table 1

Listeners' mean perceived anxiety scores and musician's self-rated anxiety

Movement	Performance condition and stimulus modality	Listeners' mean perceived anxiety scores	Pianist's self-rated anxiety	Difference	SD	95 % CI	
						Lower	Upper
Second	Practice Audio	37.95	50.00	-12.05	18.82	-17.52	-6.59
	Practice Audiovisual	32.5	57.00	-24.50	18.25	-29.80	-19.20
	Recital Audio	40.03	44.50	-4.47	15.51	-8.97	0.04
	Recital Audiovisual	30.66	60.50	-29.84	16.98	-34.77	-24.91
Third	Practice Audio	40.67	44.00	-3.33	15.16	-7.73	1.07
	Practice Audiovisual	40.94	44.50	-3.56	17.50	-8.64	1.52
	Recital Audio	40.53	67.00	-26.47	22.49	-33.00	-19.94
	Recital Audiovisual	40.86	71.50	-30.64	17.70	-35.78	-25.49

Supplementary Table 2

Extracted musical features

Piece		Duration (Seconds)	Tempo (bpm)	Pulse clarity	Attack leap
Second movement (Allegretto)	Practice	57.93	50.82*	0.30	0.16
	Recital	57.25	55.43*	0.32	0.12
Third movement (Presto)	Practice	94.11	130	0.32	0.16
	Recital	89.33	133.12	0.31	0.17

Note: *The MIRtoolbox presents some difficulties when calculating the tempo of ternary metrics in pieces like the Second movement. Original values were corrected by dividing them by 3.

5 Code

We include the MATLAB code prepared by the second author (AM) for extracting musical features and executed in Windows.

```
%create a folder and add all your audio files in it, do not add any other files
folder_dir = 'D:\Music research\Perceptual
experiment\Perceptual_experiment_stimuli_MP3' %change it to the folder's
directory
cd(folder_dir); %goes to that folder
addpath 'D:\Music research\Perceptual experiment\MIR toolbox'
%add mirtoolbox to your path
filenames = dir;
filenames = filenames(3:end,:);
samping_rate = 44100; %I assume your audio has sampling rate of 44100 which you
will see
% when running the script. If the sampling rate is different change the
% value to the corresponding one
for i = 1:length(filenames)
a = miraudio(filenames(i).name);
sample_number = mirgetdata(a);
duration_in_seconds(i) = length(sample_number)/samping_rate;
t = mirtempo(a,'Autocor');
global_tempo(i) = mirgetdata(t);
pc = mirpulseclarity(a,'Frame');
pulse_clarity(i) = nanmedian(mirgetdata(pc));
al = mirattackleap(a);
attack_leap(i) = nanmedian(mirgetdata(al));
end
t =
table(vertcat({filenames.name})',duration_in_seconds',global_tempo',pulse_clarity
',attack_leap','VariableNames',...
    {'Track Names','Duration','Tempo','Pulse Clarity','Attack Leap'});
disp(t)
writetable(t,'MyMIRVariables.csv')
```

6 Stimuli used in the study

The interested reader can access the eight stimuli used in this study upon request to the first author (ÁMCA) or through this link:
https://drive.google.com/drive/folders/1q-lPH_iQ5ri_2eSL0nDoiv47VBmz4FSL?usp=sharing
